# Accurate Measurement of the *in vivo* Ammonium Concentration in *Saccharomyces cerevisiae*

**DOI:** 10.3390/metabo6020012

**Published:** 2016-04-23

**Authors:** Hugo F. Cueto-Rojas, Reza Maleki Seifar, Angela ten Pierick, Sef J. Heijnen, Aljoscha Wahl

**Affiliations:** Cell Systems Engineering Group, Department of Biotechnology, Delft University of Technology, 2628 BC Delft, The Netherlands; h.f.cuetorojas@gmail.com (H.F.C.-R.); R.MalekiSeifar@tudelft.nl (R.M.S.); a.ten.pierick@vu.nl (A.t.P.); J.J.Heijnen@tudelft.nl (S.J.H.)

**Keywords:** intracellular ammonium, UHPLC-IDMS, metabolomics, *in vivo* quantification, rapid sampling

## Abstract

Ammonium (NH_4_^+^) is the most common N-source for yeast fermentations, and N-limitation is frequently applied to reduce growth and increase product yields. While there is significant molecular knowledge on NH_4_^+^ transport and assimilation, there have been few attempts to measure the *in vivo* concentration of this metabolite. In this article, we present a sensitive and accurate analytical method to quantify the *in vivo* intracellular ammonium concentration in *Saccharomyces* *cerevisiae* based on standard rapid sampling and metabolomics techniques. The method validation experiments required the development of a proper sample processing protocol to minimize ammonium production/consumption during biomass extraction by assessing the impact of amino acid degradation—an element that is often overlooked. The resulting cold chloroform metabolite extraction method, together with quantification using ultra high performance liquid chromatography-isotope dilution mass spectrometry (UHPLC-IDMS), was not only more sensitive than most of the existing methods but also more accurate than methods that use electrodes, enzymatic reactions, or boiling water or boiling ethanol biomass extraction because it minimized ammonium consumption/production during sampling processing and interference from other metabolites in the quantification of intracellular ammonium. Finally, our validation experiments showed that other metabolites such as pyruvate or 2-oxoglutarate (αKG) need to be extracted with cold chloroform to avoid measurements being biased by the degradation of other metabolites (e.g., amino acids).

## 1. Introduction

One of the key challenges when studying nitrogen metabolism in different biological systems *in vivo* is measuring the intracellular ammonium concentration. Accurate data on *in vivo* intracellular ammonium concentrations are needed to study the molecular mechanisms behind its transport, assimilation, and regulation, as observed by Kim *et al.* [1]. Although the transporters and reactions by which ammonium is assimilated in cells are well known [2,3,4], little is known about the intracellular ammonium concentration and its regulatory mechanisms. In relation to *Saccharomyces* *cerevisiae*, many studies have described how central nitrogen metabolism is tightly regulated by the presence of ammonium, glutamate, glutamine, and other nitrogenous compounds [4,5,6].

Interestingly, in most of the studies related to ammonium transport, not NH_4_^+^, but analogous non-metabolizable molecules were used, such as methylamine [7,8,9]. In particular, the first attempts to describe ammonium transporter proteins involved the use of ^14^C-labeled methylamine to determine the kinetics of the transporters [8]. Intracellular ammonium was not measured in these transport studies, to our knowledge. Other studies measured only the extracellular ammonium concentration and correlated this measurement with intracellular observations, or simply estimated the intracellular ammonium concentration based on certain assumptions, such as thermodynamic equilibrium of the reaction glutamate dehydrogenase [1,10]. However, there is no experimental *in vivo* evidence for those assumptions.

In earlier studies [9], measuring intracellular ammonium in *S*. *cerevisiae* for resting cells using a water-based extraction method combined with an enzymatic method; Barreto *et al.* [11] applied the same approach. On the other hand, Tate and Cooper [12] used an ammonium-specific electrode to study the relationship between intracellular ammonium and the genetic control mechanisms of the central nitrogen metabolism. Although the results obtained correlated with biological observations, it is debatable whether the N-metabolism was properly quenched and whether the leakage of intracellular ammonium was absent. For these reasons, it is not clear whether these methods for intracellular ammonium measurement are accurate where cells are growing with a high N-uptake rate, as in N-limited chemostats.

Sampling and analytical protocols for the quantitative analysis of intracellular ammonium must meet the following requirements [13]:

(1) Formation or consumption of NH_4_^+^ during sample processing must be avoided.
(2) Extensive leakage of NH_4_^+^ must be absent.
(3) Extraction of intracellular NH_4_^+^ from biomass must be fast and complete.
(4) Proper internal standards must be applied.

The aim of this article is to present an accurate and reliable analytical protocol for the quantification of intracellular ammonium in *S*. *cerevisiae*. Our methods include conventional rapid sampling as well as new methods for biomass extraction and Isotope Dilution Mass Spectrometry (IDMS)-based analysis of ammonium. All biological samples were obtained from cultivations of *S*. *cerevisiae* in aerobic chemostats using glucose as C-source under N-source limitation at pH = 5.

## 2. Results

During the method development phase, we considered different analytical techniques, namely colorimetric and enzymatic assays. However, these techniques presented major drawbacks as they were either not specific enough (colorimetric), or the sample matrix decreased the effectiveness (enzymatic). In particular, the high glutamic acid content in the intracellular samples interfered with any enzymatic assay based on the glutamate dehydrogenase reaction, since we could observe an end-product inhibition of the reaction due to thermodynamic equilibrium. Therefore, we used an ultra high performance liquid chromatography-isotope dilution mass spectrometry (UHPLC-IDMS)-based method to overcome these challenges.

### 2.1. Reproducibility and Linearity of the Method Using Standards

We prepared different calibration lines in order to validate day-to-day reproducibility using the measured ^15^N to ^14^N ratio, corrected for the influence of natural isotopes [14]. The slope is reproducible with 1.25% standard deviation, and the offset with 19% standard deviation. The correlation coefficient of the calibration line is r^2^ = 0.9967. From these results, we concluded that the method is linear in the concentration range between 1 to 750 µM.

### 2.2. Comparison between Centrifugation and Filtration for Biomass Separation

Initially, all biological samples were obtained using the filtration method described by Suarez-Mendez *et al.* [15]. After some preliminary tests using biological samples measured by UHPLC-IDMS, we observed a significant reduction (>90%) in the peak area of the internal standard (^15^N-NH_4_^+^, *m/z* = 187) between standards and biological samples. We determined the resolution of the MS to be 0.7 mass units, given that no interferences of other masses were detected at this mass difference. We therefore concluded that the instrument was not the source of such deviation between biological samples and standards. In order to isolate the cause of the reduction of peak intensities, we decided to test the extraction method under different conditions. The results and details of each set of samples are summarized in supplementary Table S1. From these tests, it was possible to infer the negative effect on peak intensities of 0.45 µm filters used in the filtration method during the ethanol-boiling step.

Although the filtration method did not work as expected, the results obtained using standards without filters were encouraging enough to continue the research using a processing method based on centrifugation for biomass separation. Indeed, further tests showed no significant changes in peak areas of labelled internal standards between standard calibration solutions and biological samples.

### 2.3. NH_3_-Evaporation during Rapid Vap Drying

NH_4_^+^ is in equilibrium with NH_3_, which is a hydrophilic gas that could escape from aqueous solution during the vacuum drying step. 120 µL of a 500 µM solution of ammonium dihydrogen phosphate was boiled together with 120 µL of internal standard (^15^N-NH_4_Cl 500 µM) in 30 mL of ethanol 75% (*v/v*), and processed following the same procedure as the typical intracellular samples [13]. After being dissolved in milli-Q water, the samples were derivatized and measured using liquid chromatography–mass spectrometry (LC-MS), as described above. The experimental results showed that NH_3_ evaporation could be corrected by adding ^15^N-NH_4_Cl to the sample as internal standard during the sample processing. Other experiments were carried out using extraction mixtures obtained from BE5 (buffered boiling ethanol), CM5 (cold-buffered chloroform-methanol extraction), and CM (cold non-buffered chloroform-methanol extraction) protocols, leading to similar results.

### 2.4. Absence of Matrix Effects

A known amount of ammonium was spiked to different samples, aiming to show that the amount measured in the spiked samples (intracellular, extracellular, or total broth samples) is the sum of the amount present in the sample plus the added amount in order to demonstrate that no matrix effect takes place during the analysis. Table 1 presents the results of the spike experiments for samples obtained after CM5 extraction; all samples were spiked with the equivalent of 50 µM NH_4_^+^. In all cases, the *t*-test (*p* < 0.05, three independent observations) showed no significant difference between spiked samples and reference samples, confirming the absence of matrix effects in the NH_4_^+^ analysis.

### 2.5. Intracellular Ammonium Quantification Requires Extraction with Cold-Buffered Methanol-Chloroform

During quenching and extraction, leakage and/or chemical conversion of NH_4_^+^ could occur. Such reactions are more common with extreme pH, high temperatures and high concentrations. Due to the different amounts of processed biomass, the resulting metabolite concentrations in the extraction mixture for intracellular (IC) samples were approximately six-fold higher than for total broth (TB) samples. Table 2 shows a balance gap of +25% for the conventional BE method, which indicates ammonium formation from other metabolites during the boiling of the biomass pellet, or consumption of NH_4_^+^ in the total broth by an unknown chemical reaction. For the buffered boiling ethanol protocol (BE5), there was a gap of −20%, showing the opposite behavior. Only for the cold-buffered chloroform-methanol extraction (CM5) protocol was there no significant gap in the total ammonium balance, which required that data reconciliation be performed in order to calculate the best estimate of the real state (Table 2). An additional observation was that the CM5 method led to the best reproducibility.

Finally, we were able to use (for the CM5 method) the constraint of the NH_4_^+^-balance to obtain the reconciled data (Table 2). Note that of the total NH_4_^+^ present in the NH_4_^+^-limited fermentation broth, only 7% is found in the extracellular space. The remaining 93% is present in the intracellular space.

### 2.6. Amino Acid Degradation

**Figure 1:**
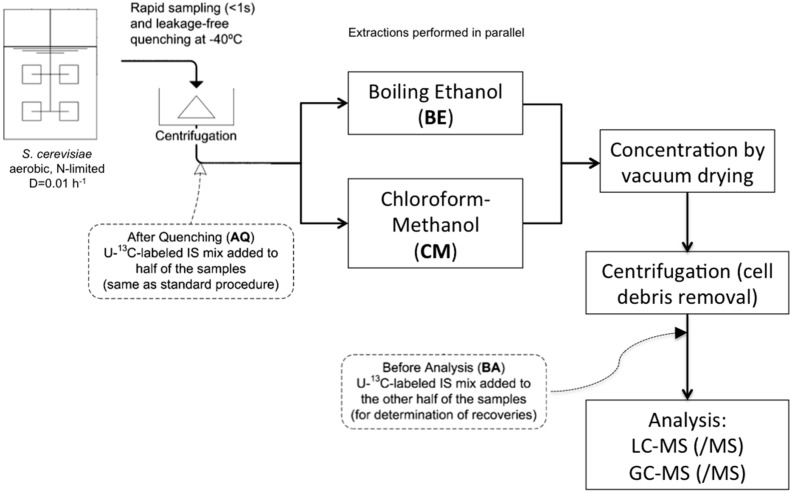
Experimental approach for quantifying amino acid degradation (adapted from [13]).

We prepared different samples for the different extraction protocols with two time-points for adding U-^13^C cell extract, as in [13] (Figure 1), in order to quantify the occurrence of amino acid degradation and NH_4_^+^ formation. Table 3 shows significant differences in BE5 after quenching and before analysis.

## 3. Discussion

The experimental results show that reactions occur involving NH_4_^+^. For instance, glutamine is a labile amino acid that degrades spontaneously to ammonium and pyroglutamic acid Equation (1).

(1) $$\mathrm{L-glutamine}\to {\mathrm{NH}}_{3}+\mathrm{Pyroglutamicacid}\;\Delta_{r}G^{0^{’}}=-30.9\frac{\mathrm{kJ}}{\mathrm{mol}}$$

A range of factors such as temperature and pH affect the kinetics of this degradation process. Furthermore, glutamic acid also decays into pyroglutamic acid in a similar manner Equation (2), but this reaction is much slower than glutamine decay and does not produce NH_3_. Moreover, due to the nature of the extraction methods (e.g., the boiling ethanol method), it is likely that some amino acid degradation or pH shifts take place.

(2) $$\mathrm{L-glutamicacid}\to H_{2}O+\mathrm{Pyroglutamicacid}\;\Delta_{r}G^{0^{’}}=-19.5\frac{\mathrm{kJ}}{\mathrm{mol}}$$

Given the very large intracellular concentration of glutamine and glutamate (in the order of 100 µmol/g_CDW_) the spontaneous decay of glutamine (which produces NH_4_^+^) can noticeably compromise the intracellular NH_4_^+^ concentration measured. L-glutamine is more stable in the pH range between pH = 5 and 7, therefore we tested buffered extraction solutions (BE5 and CM5). We expected that the cold chloroform-methanol-buffered CM5 extraction method would display the least amino acid conversion, which proved to be the case. From Table 3, we can see that there is a reduction of alanine of 1.23 µmol/gCDW, which explains the origin of the 1.75 µmol/gCDW (1.96 µmol/gCDW–0.21 µmol/gCDW) increase in pyruvate. We found other metabolites such as PEP in lower concentrations than alanine and expect their contribution to increased pyruvate concentrations to be less significant. For glutamate, there is a decrease of 1.74 µmol/gCDW, and for glutamine a decrease of 0.95 µmol/gCDW. The decrease in glutamate and glutamine is accompanied by an increase of αKG and pyroglutamate levels compared to CM5 extraction. This indicated that both glutamine and glutamate degrade to αKG and ammonium, which is particularly important since αKG concentrations are then overestimated six-fold and intracellular NH_4_^+^ is also significantly overestimated, which explains the NH_4_^+^-balance deviations observed, as discussed above.

Furthermore, we observed important differences in the levels of pyroglutamate; although small, this also suggests that glutamine or glutamate is converted into pyroglutamate during sample processing. These results show that extraction of ammonium from biomass must be carried out using the cold-buffered chloroform-methanol CM5 extraction method to avoid measurements being biased by the degradation of amino acids, given that the concentration ratio of amino acid/precursor is usually very high.

Here, we introduce a sampling/analysis method for accurately measuring intracellular NH_4_^+^. Our experimental results demonstrated that intracellular ammonium measurements require biomass separation using a centrifugation method. They also demonstrated that the standard filtration method resulted in biased measurements originated by the filters used. Centrifugation, on the other hand, enabled us to obtain reproducible samples, with no other matrix effect. We therefore selected centrifugation as the standard method. Furthermore, the experimental evidence firmly established that NH_3_-evaporation could be corrected using ^15^N-NH_4_Cl as internal standard.

In addition, the various validation tests showed that cold-buffered chloroform-methanol CM5 was the best method for extracting intracellular ammonium because it prevented formation/consumption of ammonium during sample processing. However, we advise caution when using this method to extract pH-sensitive metabolites (e.g., coenzymes). Further experimental evidence also suggested that the boiling ethanol extraction procedure degrades amino acids, particularly glutamate and glutamine, generating pyroglutamic acid. Therefore, amino acids should be extracted using cold methods, such as cold chloroform-methanol-water extraction.

## 4. Materials and Methods 

### 4.1. Strain and Culture Conditions

The biological samples used for method validation experiments were obtained from available yeast cultivations. The strain used for validation of the intracellular ammonium method was *Saccharomyces cerevisiae* CEN.PK *ath1 nth1 nth2* mat **a** MAL2-8c *leu2* *ath1∆::kanMX4 nth1∆::kanMX4 nth2∆::LEU2* [16], kindly provided by Dr. Jean-Luc Parrou from the Ingénierie des Systèmes Biologiques et des Procédés at INSA-Toulouse, France. Yeast cells from this strain were cultivated in aerobic ammonium-limited chemostat conditions in a 2 L fermentor (Applikon, Schiedam, The Netherlands) with a working volume of 1 L. The dilution rate was maintained at 0.1 h^−1^, the temperature was kept constant at 30 °C, and the pH was kept constant at a value of 5 with automatic additions of 4 M KOH or 2 M H_2_SO_4_. Dissolved oxygen tension (DOT) was monitored online using an oxygen probe (Mettler-Toledo, Tiel, The Netherlands). A stirring speed of 600 rpm, overpressure of 0.3 bar, and aeration rate of 0.25 vvm were used in order to keep the dissolved oxygen level above 40%.

The medium used was a modification of the N-limiting medium reported by Boer *et al.* [17], with the following composition: glucose 130 g/L, MgSO_4_·7H_2_O 1.14 g/L, ethanol 25 g/L (supplemented to avoid oscillations), KH_2_PO_4_ 6.9 g/L, trace elements 2 mL/L, vitamin solutions were added 2 mL/L and antifoam C 0.3 g/L; the N-source used was NH_4_H_2_PO_4_ 3.48 g/L, equivalent to 30 mM of nitrogen, which is sufficient to produce about 8 g_CDW_/L. All samples were taken at steady state, after stable values of DOT and off-gas CO_2_ and O_2_ were obtained, between three and eight volume changes after starting the feed.

### 4.2. Standards

Ammonium standards were prepared using serial dilutions of ammonium dihydrogen phosphate (NH_4_H_2_PO_4_ 5 mM) in milli-Q water. The concentrations used for the calibration lines were between 1 to 500 μM. An additional standard of 250 μM of ammonium was prepared and used as spike solutions. ^15^N-NH_4_Cl 98% atom ^15^N LOT# TA0525V (Sigma-Aldrich, Zwijndrecht, the Netherlands) was used to prepare a stock solution with a concentration of 500 µM dissolved in milli-Q water and used as internal standard in samples, as well as calibration standards.

### 4.3. Sampling and Sample Preparation

#### 4.3.1. Samples for Extracellular Metabolites and Ammonium Analysis 

Samples of approximately 1 mL broth were quenched using cold steel beads in a syringe, as described by Mashego *et al.* [18], and rapidly filtered using 0.45 μm disc filters (Millipore). 80 µL of filtrate were mixed with 20 µL of internal standard (500 µM ^15^N-NH_4_Cl) and derivatized according to the protocol used for ammonium quantification by LC-MS.

#### 4.3.2. Rapid Sampling and Biomass Extraction for Intracellular Metabolites and Intracellular Ammonium

Samples of approximately 1.2 g of broth were taken with a dedicated rapid-sampling setup [19], quenched in 6 mL of −40 °C methanol 100%, and after weighting they were centrifuged for 5 min at 10,000 *g* and −19 °C. For biomass washing, the pellet was recovered and re-suspended in 6 mL −40 °C methanol 100%, centrifuged again for 5 min at 10,000 *g* and −19 °C. For the biomass pellet extraction, four different protocols were used, because of possible production/consumption of N-containing compounds in the conventional (BE) extraction protocol.

#### 4.3.3. Boiling Ethanol Extraction of the Biomass Pellet

The biomass pellet obtained from *Rapid sampling for intracellular metabolites and intracellular ammonium* was recovered and re-suspended in 6 mL of ethanol-milli-Q 75% (*v/v*) pre-heated at 75 °C (*BE*), which is the conventional metabolite extraction for *S*. *cerevisiae* [13], or using 6 mL of ethanol 75% (*v/v*)-acetate buffer 10 mM (pH = 5) 25% (*v/v*) pre-heated at 75 °C (*BE5*) in order to extract intracellular metabolites and ammonium as described by [13]. 

120 μL of U-^13^C- cell extract (intracellular metabolites samples) or 120 μL of ^15^N- NH_4_Cl 500 µM (intracellular ammonium samples) were added as internal standard prior the addition of the buffered ethanol (AQ in Figure 1). Some samples were spiked with 120 μL NH_4_H_2_PO_4_ 250 µM; the spike solution was added at the same time as the internal standard. At this stage, all extraction mixtures were stored for a couple of days at −80 °C until further processing. 

The extraction mixture was dried using a rapidvap with cold trap (Labconco, USA) for 120 min, at a pressure lower than 5 mbar at 30 °C. The dry residue was re-suspended in 600 μL of milli-Q water and centrifuged for 5 min at 1 °C and 15,000 *g* to remove the cell debris, the supernatant was recovered in screw-cap tubes and stored at −80 °C until further processing. In some samples, 120 μL of U-^13^C- cell extract was added to the dry residue after the rapidvap-drying step (BA in Figure 1), without a previous addition of internal standard. The objective of this was to determine the extent of degradation during ethanol boiling of certain metabolites, which could lead to liberation of NH_4_^+^, such as glutamate conversion to αKG.

#### 4.3.4. CM5: Cold Chloroform-Methanol Buffered at pH = 5 Extraction of the Biomass Pellet

The biomass pellet obtained from *Rapid sampling for intracellular metabolites and intracellular ammonium* was suspended in 3.5 mL of methanol-acetate buffer 10 mM (pH of buffer solution without methanol was 5) 50% (*v/v*) pre-chilled at -40 °C. Afterwards, 3.5 mL of chloroform 100% pre-chilled at −40 °C was added followed by the addition of 120 μL of U-^13^C- cell extract (intracellular metabolites samples) or 120 μL of ^15^N- NH_4_Cl 500 µM (intracellular ammonium samples) as internal standards (AQ in Figure 1).

Selected samples were spiked with 120 μl of NH_4_Cl 250 µM. The spike solution was added at the same time with the internal standard; during the holding times, the tubes were kept at −40 ºC in a cryostat to avoid metabolic reactions. The chloroform and aqueous phases were homogenized (vortex) in order to create an emulsion (<3 s); afterwards, the tubes were kept at cold temperature (approx. −50 °C) for 45 min under constant vigorous agitation using an in-house built shaker.

After this first extraction, the tubes were centrifuged at 10,000 *g* at −20 °C for 5 min, with a rotor chilled at −50 °C; the supernatant (aqueous phase) was recovered, 3.5 mL of fresh methanol-acetate buffer 10 mM (pH = 5) 50% (*v/v*) pre-chilled at −40 °C were added; the samples were homogenized (<3 s); afterwards, the tubes were kept at cold temperature (approx. −50 ºC) for 5 min under constant vigorous agitation using an in-house built shaker. After this second extraction step, the samples were centrifuged at 10,000 *g* at −20 °C for 5 min, with a rotor chilled at −50 °C; the supernatant was again recovered and pooled together with the supernatant of the first extraction. All supernatants were stored at −80 °C until further processing.

The pooled supernatant was dried using a rapidvap with cold trap (Labconco, USA) for 180 min, at a pressure lower than 5 mbar at 30 °C. The dry residue was re-suspended in 600 μL of milli-Q water and centrifuged for 5 min at 1 °C and 15,000 *g* to remove the cell debris, the supernatant was recovered in screw-cap tubes and stored at −80 °C until further processing. In some samples, 120 μL of U-^13^C- cell extract was added to the dry residue after the rapidvap-drying step (BA in Figure 1), without a previous addition of internal standard; the objective of this was to determine the extent of degradation of certain N-containing metabolites, as explained for the BE5 extraction protocol.

#### 4.3.5. CM: Cold Non-Buffered Chloroform-Methanol Extraction of the Biomass Pellet

The biomass pellet obtained from *Rapid sampling for intracellular metabolites and intracellular ammonium* was recovered, 3.5 mL of Methanol-milli-Q water 50%(*v/v*) pre-chilled at −40 °C was added, and then 120 μL of U-^13^C- cell extract. Afterwards, 3.5 mL of Chloroform 100% pre-chilled at −40 °C was added in order to extract intracellular metabolites according to [13]. This method was only used to extract acid-unstable intracellular metabolites (e.g., NADH and NADPH); it was not used for extraction of intracellular ammonium.

Also for total broth analysis, three different extraction protocols were used, for the same reasons as explained in the methods for biomass pellet extraction.

#### 4.3.6. TBE5: Boiling Buffered Ethanol pH = 5 Extraction of Total Broth

Approximately 1.2 g of broth were taken from the reactor with a dedicated rapid-sampling setup [19], quenched in 6 mL of −40 °C methanol 100% and weighted to determine the exact mass of the sample. 3.5 mL of quenched broth solution were further processed; in all steps, the sample was weighted to determine its exact mass. 120 μL of U-^13^C- cell extract or 120 μL of ^15^N- NH_4_Cl 500 µM (intracellular ammonium samples) was added as internal standard. 3.5 mL of quenched broth/methanol solution were boiled in 30 mL of ethanol-acetate buffer 10 mM (pH = 5) 75% (*v/v*) pre-heated at 75 °C as described by [13].

Subsequently, the extraction mixture was dried using a rapidvap with coldtrap (Labconco, USA) for 240 min, at a pressure lower than 5 mbar at room temperature. The dry residue was re-suspended in 600 μL of milli-Q water, centrifuged for 5 min at 1 °C and 15,000 *g* to remove the cell debris. The supernatant was recovered in screw-cap tubes and stored at −80 °C until further processing.

#### 4.3.7. TBE: Boiling Non-Buffered Ethanol Extraction of Total Broth

Approximately 1.2 g of broth were taken from the reactor with a dedicated rapid-sampling setup [19], quenched in 6 mL of −40 °C methanol 100% and weighted to determine the exact mass of the sample. 3.5 mL of quenched broth solution were further processed; in all steps, the sample was weighted to determine its exact mass. 120 μL of U-^13^C- cell extract or 120 μL of ^15^N- NH_4_Cl 500 μM (intracellular ammonium samples) was added as internal standard. The sample was boiled in 30 mL of ethanol 75% (*v/v*) in milli-Q water pre-heated at 75 °C as described above.

#### 4.3.8. TCM5: Cold Chloroform-Methanol Buffered at pH = 5 Extraction of Total Broth

Approximately 1.2 g of broth were taken from the reactor with a dedicated rapid-sampling setup [19], quenched in 6 mL of −40 °C methanol-acetate buffer pH = 5 60% (*v/v*), and weighted to determine the exact mass of the sample. 3.5 mL of quenched broth solution were further processed; in all steps the sample was weighted to determine its exact mass. 120 μL of U-^13^C- cell extract or 120 μL of ^15^N-NH_4_Cl 500 µM (intracellular ammonium samples) was added as internal standard, followed by addition of chloroform 100% pre-chilled at −40 °C for extraction using the CM5 protocol for intracellular samples; the pooled supernatants were dried using a rapidvap with cold trap for 180 min, at a pressure lower than 5 mbar at 30 °C. The dry residue was re-suspended in 600 μL of milli-Q water and centrifuged for 5 min at 1 °C and 15,000 *g* to remove the cell debris, the supernatant was recovered in screw-cap tubes and stored at -80 °C until further processing.

### 4.4. Analytical Methods

#### 4.4.1. Ammonium Quantification 

The ammonium in the samples was quantified using UHPLC-IDMS after derivatization of the sample with Diethyl Ethoxymethylenemalonate (DEEMM) as described by Redruello *et al.* [20]. Briefly, 100 μL of solution to be analyzed obtained from intracellular, extracellular or total broth samples were mixed with 175 μL of Na_3_BO_3_ buffer 1 M (pH = 9), 75 μL of 100% methanol, 3 μL of DEEMM, in glass vials. The vials were incubated at 30 °C for 45 min to complete the derivatization reaction; excess DEEMM was degraded by incubation at 70 °C for 2 h. All measurements were performed on an AcQuity^TM^ UPLC system (Waters, Milford, MA, USA) coupled to a Quattro Premier XE mass spectrometer (Micromass MS Technologies-Waters, Milford, MA, USA) with an electrospray ion source. The MS was operated in negative mode. Masslynx 4.1 (Waters) used for data acquisition and peak integration.

Metabolite detection was performed in selected ion monitoring mode (SIM). The general settings were as follows: the ESI capillary voltage was −2.8 kV, extractor voltage 5 V, RF lens voltage 0.5 V, and cone voltage 35 V. The desolvation gas (nitrogen) flow was 700 L/h at 360 °C, the cone gas (nitrogen) flow was 50 L/h, and the source block temperature was set at 120 °C. For ^14^N-aminoene the *m/z* of 186 and for ^15^N-aminoene the *m/z* of 187 were monitored. The peak areas (heights) of internal standards were corrected considering blank injections and natural isotope contributions from carbon, nitrogen, hydrogen, and oxygen atoms of the derivatization reagent. The chromatographic separation of ammonium was adopted using the protocol described by Maleki Seifar *et al.* [21].

#### 4.4.2. Metabolite Quantification 

Quantification of αKG, pyruvate, and trehalose was performed using GC-MS/MS as described by Niedenfuhr *et al*. [14]. Amino acids were measured using GC-MS according to de Jonge *et al.* [22].

### 4.5. Data Reconciliation

Data reconciliation was performed using the total broth mass balance ( [TB] = [IC] × gCDW/L_TB_ + [EC]) and the experimental measurements according to Verheijen [23], under the constraint that mass conservation is satisfied. 

## Figures and Tables

**Table 1:** Results of the spike experiments using cold-buffered chloroform-methanol extraction (CM5) as extraction method. The estimated concentration when the spiked amount (50 µM) is subtracted from the measurement is shown in brackets. The results are displayed as µM_sample_. The results are single injections of independent triplicates.

Sample	Measured Concentration µM_sample_ (measurement-spike)	Average ± St. Dev. µM_sample_
**Extracellular 1 (Oct 14)**	6.1	4.2 ± 2.8
**Extracellular 2 (Oct 14)**	2.2	
**Extracellular 3 (Oct 14)**	N.D.	
**Spiked extracellular 1 (Oct 14)**	61.1 (11.1)	9.2 ± 2.1 ^1^
**Spiked extracellular 2 (Oct 14)**	57.0 (7.0)	
**Spiked extracellular 3 (Oct 14)**	59.5 (9.5)	
**Intracellular 4 (Oct 14)**	35.9	31.2 ± 4.1
**Intracellular 5 (Oct 14)**	28.7	
**Intracellular 6 (Oct 14)**	28.9	
**Spiked intracellular 4 (Oct 14)**	94.4 (44.4)	34.3 ± 9.0 ^1^
**Spiked intracellular 5 (Oct 14)**	81.3 (31.3)	
**Spiked intracellular 6 (Oct 14)**	77.2 (27.2)	
**Total broth 4 (Oct 14)**	21.8	22.7 ± 1.3
**Total broth 5 (Oct 14)**	22.1	
**Total broth 6 (Oct 14)**	24.2	
**Spiked total broth 4 (Oct 14)**	77.8 (27.8)	29.1 ± 6.5 ^1^
**Spiked total broth 5 (Oct 14)**	73.3 (23.3)	
**Spiked total broth 6 (Oct 14)**	86.2 (36.2)	

^1^ Average of estimated concentration (measured concentration–spiked amount).

**Table 2:** Results of total broth ammonium mass balance. Reconciled concentrations provide the best estimates of the measurements obtained by least square minimization of the differences between the measurements and estimated amounts, weighted with respect to their measurement errors.

Type of Sample	Concentration Measured ^1^ (µM_broth_)	Recovery (%)	Reconciled Concentration * (µM_broth_)
Intracellular BE (Aug 14)	24.51 ± 2.15	124.50%	N.A.
Extracellular (Aug 14)	6.23 ± 1.15		
Total broth BE (Aug 14)	24.69 ± 5.25		
Intracellular BE5 (Oct 14)	10.04 ± 1.93	77.93%	N.A.
Extracellular (Oct 14)	1.05 ± 0.49		
Total broth BE5 (Oct 14)	14.23 ± 0.23		
Intracellular CM5 (Oct 14)	15.16 ± 1.24	96.66%	15.57 ± 0.64
Extracellular (Oct 14)	1.05 ± 0.49		1.12 ± 0.46
Total broth CM5 (Oct 14)	16.77 ± 0.57		16.68 ± 0.52

^1^ Average of three independent samples injected and quantified three times; N.A. = not applicable. BE: conventional boiling ethanol extraction; BE5: buffered boiling ethanol protocol.

**Table 3:** Quantitative analysis of amino acid decay using different extraction methods. The results displayed are averages of three independent samples (in µmol/gCDW).

^13^C Addition	Extraction Solution	Intracellular Metabolite Concentration (µmol/gCDW) (% Recovery with Respect to ^13^C Addition after Quenching)							
		Ala	Glu	Asp	Gln	PyrGlu	Pyruvate	αKG	NH_4_^+^
AQ	BE5	6.97 ± 0.21	52.79 ± 0.36	11.51 ± 0.14	10.77 ± 0.25	0.78 ± 0.02	1.96 ± 0.17	3.01 ± 0.05	3.46 ± 0.28
BA	BE5	5.73 ± 0.25 (82.3%) ^1^	51.05 ± 0.75 (96.7%)	10.80 ± 0.33 (93.9%)	9.81 ± 0.19 (91.2%)	1.11 ± 0.02 (141.9%) ^1^	N.M.	N.M.	N.M.
AQ	CM5	6.50 ± 0.07	51.95 ± 0.65	9.03 ± 0.09 ^2^	10.85 ± 0.16	0.51 ± 0.02 ^2^	0.21 ± 0.01	0.50 ± 0.01	2.29 ± 0.44
BA	CM5	6.00 ± 0.05 (92.4%) ^1^	49.95 ± 0.30 (96.2%)	8.90 ± 0.07 (98.5%)	10.68 ± 0.07 (98.4%)	0.57 ± 0.06 (112.2%)	N.M.	N.M.	N.M.
AQ	BE	7.86 ± 0.13	56.00 ± 0.95	11.54 ± 0.28	11.28 ± 0.14	N.M.	2.17 ± 0.24	2.81 ± 0.06	5.78 ± 0.20

gCDW = gram of cell dry weight. N.M. = not measured. In these cases, the analysis was not performed, therefore no experimental data are available; ^1^ Statistically significant difference found between AQ and BA samples, according to a *t*-test at a significance level of 0.05. ^2^ Statistically significant difference found between BE5 AQ and CM5 AQ samples, according to a *t*-test at a significance level of 0.05.

## Supplementary Materials

The following are available online at http://www.mdpi.com/2218-1989/6/2/12/s1, Table S1: Comparison between samples exposed to PVDF filters and samples not exposed to PVDF filters.

## Abbreviations

The following abbreviations are used in this manuscript:

αKG: 2-Oxoglutarate
AQ: After quenching
BA: Before analysis
BE: Boiling ethanol intracellular metabolite extraction (non-buffered)
BE5: Buffered (pH = 5) boiling ethanol intracellular metabolite extraction
CM: Cold chloroform-methanol-water intracellular metabolite extraction (non-buffered)
CM5: Cold-buffered (pH = 5) chloroform-methanol-water intracellular metabolite extraction
DEEMM: Diethyl ethoxymethylenemalonate
DOT: Dissolved oxygen tension
IC: Intracellular
IDMS: Isotope dilution mass spectrometry
PEP: Phosphoenolpyruvate
SIM: Selected ion monitoring
TB: Total broth
TBE: Boiling non-buffered ethanol extraction of total broth
TBE5: Boiling buffered (pH = 5) ethanol extraction of total broth
TCM5: Cold chloroform-methanol buffered (pH = 5) extraction of total broth
UHPLC-IDMS: Ultra high performance liquid chromatography-isotope dilution mass spectrometry
