# Supplementary material for: Accurate Measurement of the in vivo Ammonium Concentration in Saccharomyces cerevisiae
Source: Metabolites. 2016 Apr 23;6(2):12. doi: 10.3390/metabo6020012 (PMC4931543; doi:10.3390/metabo6020012)
Supplement: Supplementary file 1 [file metabolites-06-00012-s001.pdf]

Supplementary Material

Table S1. Comparison between samples exposed to Polyvinylidene Difluoride (PVDF) filters and samples not exposed to PVDF filters.

Sample set	Measured concentration* (μmol/L)	Condition
Standard 100 μL	103.6 ± 4.9	Standard solution mixed with internal standard and derivatized with DEEMM.
Centrifugation "mock" sample	105.8 ± 4.2	120 μL standard solution 500 μM NH_4^+ mixed with 120 μL internal standard in 5 mL ethanol 75% (*v/v*), boiled for 3 minutes. Re-suspended in 600 μL milli-Q after ethanol evaporation.
Standard 100 μL in 30 mL Ethanol	92.3 ± 14.7	120 μL standard solution 500 μM NH_4^+ mixed with 120 μL internal standard in 30 mL ethanol 75% (*v/v*), boiled for 3 minutes. Re-suspended in 600 μL milli-Q after ethanol evaporation.
Filtration "mock" sample	120.6 ± 20.9	120 μL standard solution 500 μM NH_4^+ mixed with 120 μL internal standard in 30 mL ethanol 75% (*v/v*), boiled together with a 0.45 μm PVDF filter for 3 minutes. Re-suspended in 600 μL milli-Q after ethanol evaporation.
Filter + NaCl in ethanol	156.3 ± 15.7	120 μL standard solution 500 μM NH_4^+ mixed with 120 μL internal standard and 100 μL of NaCl 100 mg/mL in 30 mL ethanol 75% (*v/v*), boiled together with a PVDF filter for 3 minutes. Re-suspended in 600 μL milli-Q after ethanol evaporation.
Filter + NaCl in quenching solution	117.0 ± 41.7	120 μL standard solution 500 μM NH_4^+ mixed with 120 μL internal standard in 30 mL ethanol 75% (*v/v*), boiled together with a PVDF filter for 3 minutes. The filter was previously exposed to 30 mL of methanol at -40 ºC and 100 μL NaCl 100 mg/mL. Re-suspended in 600 μL milli-Q after ethanol evaporation.
Filter incubated in DBAA	107.7 ± 33.7	120 μL standard solution 500 μM NH_4^+ mixed with 120 μL internal standard and 100 μL of NaCl 100 mg/mL in 30 mL ethanol 75% (*v/v*), boiled together with a PVDF filter for 3 minutes. The filter was previously exposed to 30 mL of methanol at -40 ºC and incubated 5 minutes in DBAA 25 mM. Re-suspended in 600 μL milli-Q after Ethanol evaporation.

* Average value ± Standard deviation of three injections of triplicate samples.
